# Expertise effects in cutaneous wind perception

**DOI:** 10.3758/s13414-015-0893-6

**Published:** 2015-04-21

**Authors:** Joost P. Pluijms, Rouwen Cañal-Bruland, Wouter M. Bergmann Tiest, Fabian A. Mulder, Geert J. P. Savelsbergh

**Affiliations:** MOVE Research Institute Amsterdam, Faculty of Human Movement Sciences, VU University Amsterdam, Van der Boechorststraat 9, 1081 BT Amsterdam, The Netherlands; Faculty of Industrial Design Engineering, Delft University of Technology, Landbergstraat 15, Delft, 2628 CE The Netherlands

**Keywords:** Wind, Perception, Expertise, Sailing, Psychophysics

## Abstract

We examined whether expertise effects are present in cutaneous wind perception. To this end, we presented wind stimuli consisting of different wind directions and speeds in a wind simulator. The wind simulator generated wind stimuli from 16 directions and with three speeds by means of eight automotive wind fans. Participants were asked to judge cutaneously perceived wind directions and speeds without having access to any visual or auditory information. Expert sailors (*n* = 6), trained to make the most effective use of wind characteristics, were compared to less-skilled sailors (*n* = 6) and to a group of nonsailors (*n* = 6). The results indicated that expert sailors outperformed nonsailors in perceiving wind direction (i.e., smaller mean signed errors) when presented with low wind speeds. This suggests that expert sailors are more sensitive in picking up differences in wind direction, particularly when confronted with low wind speeds that demand higher sensitivity.

In many domains, experts distinguish themselves from novices by their superior performance. These domains include motor performance in sports (e.g., Starkes & Ericsson, 2003) or music (e.g., Ericsson, Krampe, & Tesch-Römer, 1993), cognitive functions such as problem-solving skills (e.g., Sweller, 1988) and memory (e.g., Ericsson & Kintsch, 1995), and perceptual performance such as superior visual information pickup in car driving (e.g., Falkmer & Gregersen, 2005). As concerns experts’ perceptual superiority, research has provided ample evidence that expertise effects exist for the various sensory modalities. For example, it is well established that experts show superior visual perception (Mann, Williams, Ward, & Janelle, 2007), auditory perception (e.g., Koelsch, Schröger, & Tervaniemi, 1999), and olfactory perception (e.g., Parr, Heatherbell, & White, 2002) when compared to novices. Despite the growing knowledge of expertise effects in several sensory modalities, relatively little is known about the impact of expertise on haptic perception, and in particular, cutaneous perception by means of touch receptors within the skin (for a review, see Lederman & Klatzky, 2009).

Studies on cutaneous perception have thus far mainly focused on examining the mechanisms underlying, for instance, the cutaneous perception of pain (e.g., Chapman & Jones, 1944; Sheffield, Biles, Orom, Maixner, & Sheps, 2000), tactile sensibility (e.g., Lederman & Klatzky, 2009; Vallbo & Johansson, 1984), or the cutaneous perception of heat (e.g., Casey, Minoshima, Morrow, & Koeppe, 1996), and on developing haptic interfaces (e.g., Gurocak, Jayaram, Parrish, & Jayaram, 2003; Kulkarni, Fisher, Pardyjak, Minor, & Hollerbach, 2009). However, research dedicated to the examination of expertise effects in cutaneous perception is lacking. If one sets out to examine expertise differences in cutaneous perception, it is first necessary to identify an appropriate population in which expertise effects in cutaneous perception ought be expected or might be observable.

Which target group might provide a suitable test bed for such an enterprise? Here we suggest that expert sailors provide an excellent opportunity to examine expertise effects on cutaneous perception, because they are specifically trained to perceive wind directions and speeds as accurately as possible in order to perform at their best (for a review of the influences of wind, physiological variables, and sailing expertise, see Allen & De Jong, 2006). Early evidence supporting this assumption stemmed from work by Simonnet, Guinard, and Tisseau (2005), who examined sailing performance of blind and blindfolded sailors who were instructed to steer a rectilinear trajectory in a sailboat. For each participant, GPS tracks were compared among three conditions: powered by engine only, by engine and wind (i.e., using the sails), or by wind only. The results indicated that participants without vision sailed with fewer errors (i.e., sailing in a straight line) when wind was available. This finding is in agreement with Araújo, Davids, and Serpa (2005), who—using a computer-simulated regatta—demonstrated that expert sailors showed a higher probability of exploiting the available wind information. Finally, in a recent attempt to validate a wind simulator for virtual sailing experiments in the laboratory, Verlinden et al. (2013) found that sailors (with a minimum of 50 h sailing experience) reported the virtual sailing experience to be more realistic when wind information was available than when no wind information was provided. Though these researchers did not examine sailing performance per se, but rather used questionnaires to get insights into the representativeness of the virtual sailing experience in the simulator, the initial results lend further support to our assumption that the cutaneous perception of wind plays a crucial role in sailing. Overall, it seems justified to argue that the cutaneous perception of wind is used to facilitate, and hence optimize, performance in sailing, and thus may allow us to differentiate between different levels of expertise in sailing.

With the aim to examine expertise effects in cutaneous wind perception in the present study, we presented three groups of participants (i.e., expert sailors, less skilled sailors, and inexperienced controls) with wind stimuli reflecting 16 different wind directions and three different speeds generated by a wind simulator. Participants rated the wind directions and speeds on the basis of their cutaneously perceived wind sensations in the absence of any auditory or visual information.

## Method

### Participants

Eighteen participants (13 male, five female) volunteered to take part in the experiment. Six expert sailors (mean age 28 ± 15 years), six intermediate sailors (mean age 27 ± 12 years), and six nonsailors (mean age 35 ± 11 years) participated in the wind direction and speed estimation test. Expert sailors were recruited via the Royal Netherlands Yachting Union (KNWV) in cooperation with InnoSportLab, The Hague. The expert sailors (mean sailing experience 14 ± 11 years) either sailed in the highest Dutch squad (professional level) or were members of the national youth teams of The Netherlands (talent teams; under 21 years of age); all competed at an international level. The intermediate sailors (mean sailing experience 17 ± 13 years) were defined as all sailors without sailing experience at an international level. The nonsailors were participants with no sailing experience. The ethics committee of the local institution (Faculty of Human Movement Sciences, VU University Amsterdam, The Netherlands) approved the experiment, and participants gave written consent before participation.

### Apparatus and stimulus production

**Fig. 1:**
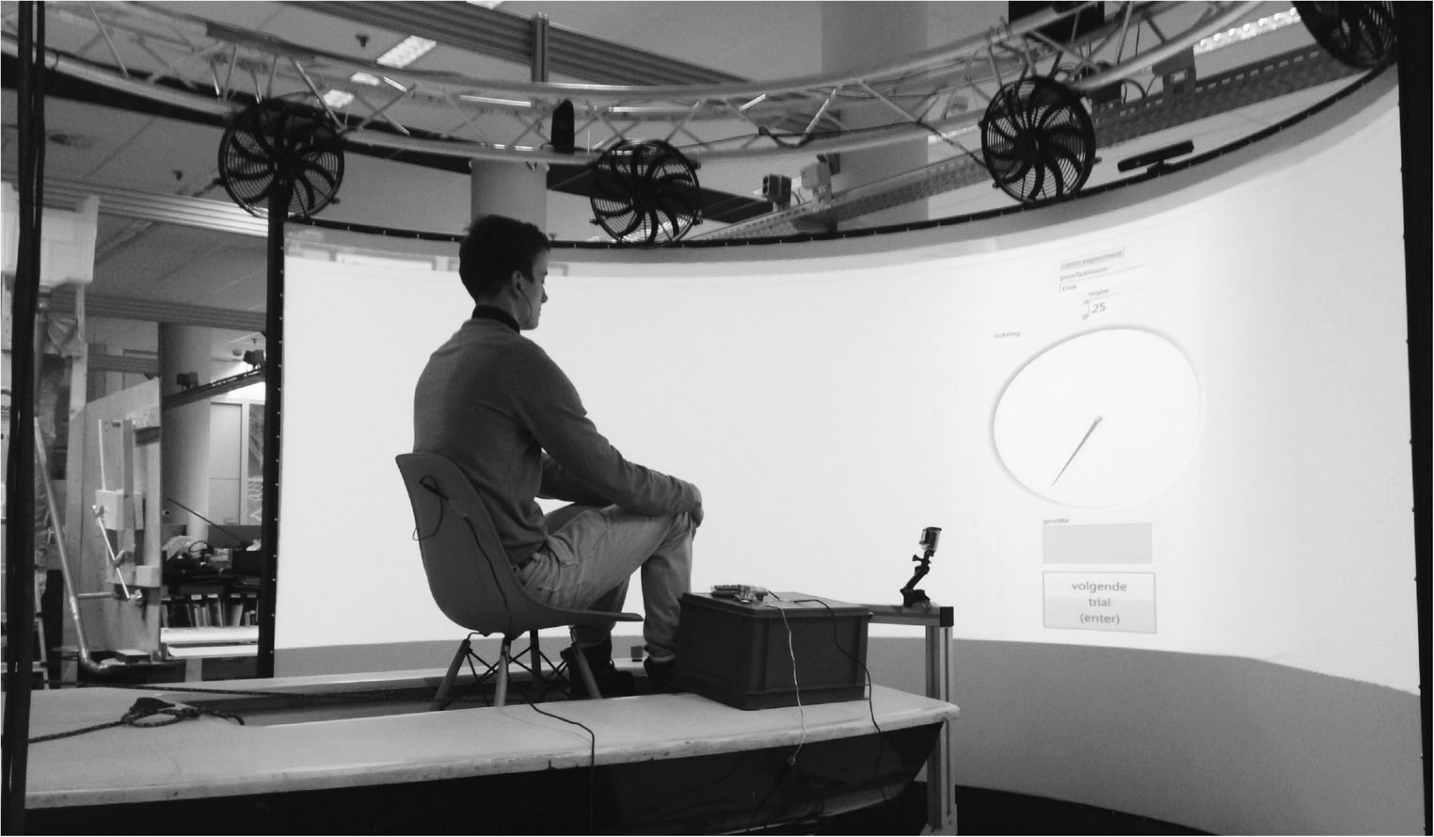
Automotive wind fans were attached to an octagonal ring, and participants were seated in the center of the octagonal ring while facing a video screen in front of them. The details of the video screen are illustrated in Fig. 3

A wind simulator integrated within a sailing simulator (Faculty of Industrial Design Engineering, Delft University of Technology) was used (Mulder & Verlinden, 2013; Mulder, Verlinden, & Dukalski, 2012; Verlinden et al., 2013). The wind direction and speed were generated using eight automotive wind fans attached to an octagonal ring (4 m diameter) 2 m above ground level (shown in Fig. 1).

**Fig. 2:**
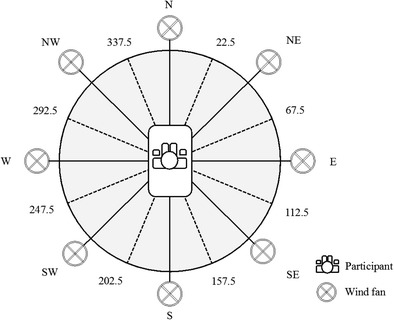
Top view of the wind simulator, including the 16 nominal wind directions (steps of 22.5°) and locations of the eight wind fans. The wind simulator was integrated within a sailing simulator. For the sailing simulator, a real boat, called the *laser dinghy*, was put in the lab. Participants were seated in the laser dinghy exactly in the middle of the octagonal ring (modified from Verlinden et al., 2013)

Each wind fan had a diameter of 40 cm and could produce a steady airflow rate between 3 and 6 knots (1.5–3 m/s) in the center of the ring. The fans were positioned at the middle of each side of the octagon and were aimed down at the participant, seated in the center, at an angle of 45 deg. The wind fans were controlled with pulse-width modulation (PWM) using microcontrollers (Arduino) and LabVIEW (National Instruments). In pulse-width modulation, the power supplied to the wind fans is rapidly switched on and off with different ratios of on- and off-times, effectively controlling the fan speed on a continuous scale. The eight wind fans could be activated alone or in pairs (next to each other), which resulted in 16 wind directions. That is, activating each single wind fan generated eight wind directions, and activating two wind fans next to each other, simultaneously and at the same speed, generated eight intermediate wind directions (e.g., activating the N and NE wind fans generated a wind direction of NNE; see Fig. 2).

Following pilot tests using an anemometer (Tacktick) and software (Pi Toolbox, Cosworth Electronics), we calculated whether various wind directions and wind speeds could be accurately simulated. The average wind direction error was 7° ± 5° for the aforementioned 16 nominal wind directions (equally distributed over 360°). To determine equal wind speeds for each wind direction, we used pulse-width modulation during the calibration tests. We tested a broad range of pulsing signals to either a single or two wind fans, to ensure equal wind speeds for all 16 wind directions. That is, the modulation differed when a single fan was used or when two wind fans were simultaneously controlled next to each other; see Fig. 2 for all wind directions). We selected three wind speed conditions after the calibration tests for each wind direction (total of 16) on the basis of the steady airflow rate for each wind fan (3–6 knots)—that is, a low (3.0 ± 0.9 knots), a medium (3.9 ± 0.9 knots), and a high (4.8 ± 1.1 knots) wind speed condition (see Table 1 and Fig. 2). Subsequent to the calibration tests, we used the actual, measured wind directions and wind speeds for further data analysis.

**Table 1:** The 16 nominal wind directions, with the corresponding compass directions (CD), mean actual angles (in degrees), and differences (in degrees) after calibration tests

Nominal Direction (°)	CD	Actual Direction (°)	Difference (°)
0	N	11.6	11.6
22.5	NNE	39.9	17.4
45.0	NE	47.5	2.5
67.5	ENE	75.9	8.4
90.0	E	95.5	5.5
112.5	ESE	124.1	11.6
135.0	SE	137.7	2.7
157.5	SSE	163.6	6.1
180.0	S	186.2	6.2
202.5	SSW	214.9	12.4
225.0	SW	234.0	9.0
247.5	WSW	240.8	–6.7
270.0	W	274.2	4.2
292.5	WNW	300.3	7.8
315.0	NW	319.7	4.7
337.5	NNW	348.2	10.7
**Average**			**7.1**
**Standard deviation**			**5.4**

### Procedure

Participants were informed that they would be presented with two blocks of 72 wind trials. They were told that the 144 randomized trials (i.e., 16 wind directions × 3 wind speeds × 3 repetitions) differed in wind direction and speed. No information was provided concerning the frequency and distribution of wind direction and speed. Participants were tested individually and seated in the center of the octagonal ring. They were instructed to face a video screen in front of them during the entire experiment. Finally, they were told that the best performance would be rewarded with a smartphone wind meter (Vaavud), to stimulate commitment throughout the experiment.

**Fig. 3:**
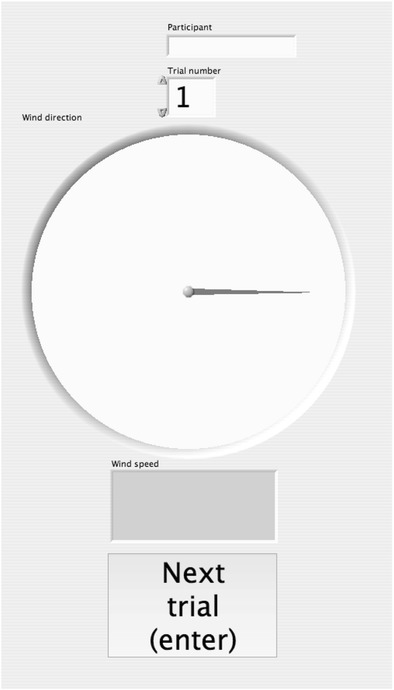
A screenshot of the video screen was presented in front of the participant (including a circle with a pointer representing wind direction)

The experiment started when a participant pressed the Enter key on a numeric keypad; additionally, participants were instructed to immediately close their eyes after pressing this key. Moreover, the participants were told that a video camera positioned right in front of them would record whether their eyes were closed (see Fig. 1). Subsequent to participants pressing the Enter key, a random wind stimulus started with a delay of 2 s (to ensure that participants had time to close their eyes) and lasted 10 s. To rule out interference from auditory information emanating from the fans, the participants were equipped with ear buds playing white noise from the start of each trial (i.e., after pressing the Enter key). The noise continued for 6 s after the wind stimulus stopped; that is, the wind fans needed 5 s at most to stop rotating. Participants were instructed to open their eyes again when the white noise stopped. Subsequently, participants could estimate the wind direction by turning a knob (Griffin Technology, PowerMate), and the wind speed by pressing the buttons on a numeric keypad. The responses (i.e., wind direction and speed) were displayed on a video screen in front of the participant (see Fig. 3). The displayed circle on which participants indicated wind direction using a pointer was shown without a scale (i.e., degrees or angles), as opposed to a compass. Participants were instructed to estimate the wind direction by turning the knob representing the pointer (following the wind stimulus that they had just cutaneously perceived). That is, participants were not asked to respond in degrees or to translate a judgment of degrees on a compass-like display.

For the wind speed estimates, a free-magnitude estimation procedure was used (Gescheider, 1997; Stevens, 1957). The only requirement for all wind speed responses was that the ratio between perception and the numerical response was preserved. After the participant pressed the Enter key again, a new trial started, and the above-mentioned procedure was repeated. Each participant practiced three example trials in order to get familiar with the procedure. A 5-min break was inserted between blocks. The length of a wind trial was approximately 30 s, and the entire experiment lasted approximately 80 min per participant.

### Data analysis

First, we calculated the mean signed error (MSE) in degrees for the wind direction estimates. We derived each MSE by subtracting the actual, measured wind direction from the wind direction estimate, allowing for a maximum error of 180°. Hence, the MSEs summarized how well the estimate matched the actual wind direction, on the basis of the calibration. The MSE is a measure of accuracy—that is, the average deviation from the actual wind direction. MSEs can be positive or negative; that is, positive errors indicated clockwise deviations, and negative errors indicated counterclockwise deviations. Note that we also analyzed and report polar plots of the mean absolute errors (see Appendix A). Next, we normalized the mean estimates for wind speed, since participants used different scales for their wind speed responses. For each trial, the normalized wind speed estimate was defined as the wind speed estimate for each separate trial (per participant), divided by the average estimate based on all 144 trials per participant. Then the estimates of all 144 trials (i.e., 144 for wind direction and 144 for wind speed) were averaged over the three randomized repetitions for each combined wind direction (16 in total) and wind speed condition (three in total), which resulted in 48 mean estimates for wind direction and 48 mean estimates for wind speed per participant.

To examine the estimates of wind direction, we performed a mixed-design analysis of variance (ANOVA) on the MSEs (in degrees), with Wind Direction (16 directions; see Table 1 and Fig. 2) and Wind Speed (low, medium, and high) as within-subjects factors, and Group (nonsailors and intermediate and expert sailors) as the between-subjects factor. Similarly, to examine the estimates of wind speed, we also performed a mixed-design ANOVA on the mean normalized estimates for wind speed, with Wind Direction (16 directions) and Wind Speed (low, medium, high) as within-subjects factors, and Group (nonsailors, intermediate and expert sailors) as the between-subjects factor. When appropriate, we performed post-hoc comparisons using Bonferroni correction. The uncorrected *α* level for significance was set at .05. Effect sizes were calculated as partial-eta-squared values (*η*_p_^2^).

Finally, we examined the relationship between the normalized estimates and actual wind speed, to investigate whether this was linear, progressive, or flattening out. Since a power function relationship is often found for magnitude estimation data (Stevens, 1957), we fitted a function of the form *f*(*x*) = *c x*^*α*^ to the normalized wind speed estimates. That is, for each participant separately (144 trials) and for the means from each group (144 data points averaged over the six participants per group), the normalized wind speed estimates were plotted against the actual wind speeds of each trial measured during calibration, which were spread out around the nominal values. As regards the function, *x* is the nominal wind speed, and *α* and *c* are free fitting parameters. The fitted exponent *α* was compared to a value of 1 (i.e., a linear relationship) using a *t* test. We also performed an ANOVA to check for group differences regarding the magnitudes of the exponents.

## Results

### Wind direction estimates

**Fig. 4:**
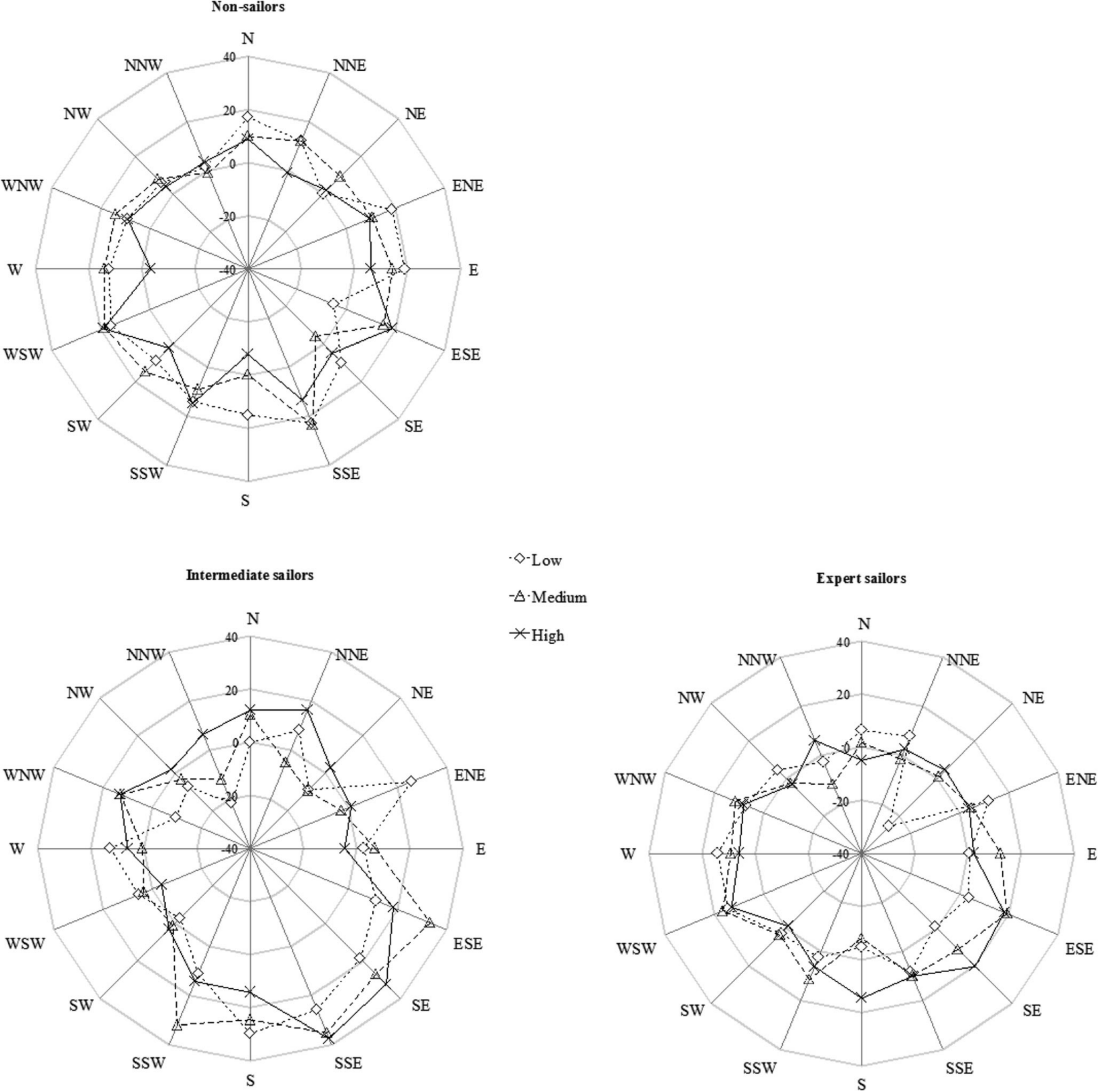
Mean signed errors (MSEs), in degrees, for the three wind speed conditions (low, medium, high) and 16 compass directions (see Table 1 for the corresponding actual wind directions); positive errors indicate overestimations (clockwise), and negative errors indicate underestimations (counterclockwise). Upper left: Nonsailors. Bottom left: Intermediate sailors. Bottom right: Expert sailors

The descriptive data (MSEs) are illustrated in Fig. 4 as polar plots for each group separately (for the polar plots of the mean absolute errors, see Appendix A).

#### Main effects

The 16 (wind directions) × 3 (wind speed: low, medium, high) × 3 (group: nonsailors, intermediate and expert sailors) mixed-design ANOVA revealed a significant main effect for wind direction on the MSEs, *F*(15, 225) = 3.3, *p* < .001, *η*_p_^2^ = .18. Further exploration of the data (see Table 2) indicated that participants by and large performed with smaller errors for frontal than for rear wind directions. We observed neither a significant main effect of wind speed, *F*(2, 30) = 1.1, *p* = *.*34, *η*_p_^2^ = .07, nor a significant main effect of group, *F*(2, 15) = 2.0, *p* = *.*17, *η*_p_^2^ = .21.

**Table 2:** Significant post-hoc pairwise comparisons, Bonferroni corrected, on the mean signed errors (MSE) for the wind direction estimates, with the 16 Compass Directions (CD) and three Wind Speed Conditions (WSC) as within-subjects factors

WSC	CD	MSE (°)	CD	MSE (°)	*p*	95% CI **for difference**
**Wind Direction**						
	ESE	14.75	NNW	–3.50	.02	1.87–34.63
	SSE	20.73	SW	3.62	.01	2.55–31.67
	SSE	20.73	NW	0.90	.03	1.07–38.59
	SSE	20.73	NNW	–3.50	.01	4.04–44.43
	WNW	8.36	NNW	–3.50	.03	0.53–23.19
**Wind Direction × Wind Speed**						
L	SSE	18.92	NNW	–7.59	.01	3.39–49.63
L	W	13.08	NNW	–7.59	<.01	6.71–34.64
M	ESE	22.60	S	5.47	.04	0.35–33.92
M	ESE	22.60	NW	0.74	.04	0.61–43.11
M	ESE	22.60	NNW	–0.85	.01	4.93–56.66
M	SSE	22.95	NNW	–0.85	.01	4.42–57.87
M	WNW	12.74	NNW	–0.85	.02	2.31–39.56
H	SSE	20.32	SW	1.38	.01	2.66–35.22
**Group × Wind Speed**						
WSC	Group	MSE (°)	Group	MSE (°)	*p*	95% CI
L	NS	11.08	ES	3.2	.03	0.75–14.96

*L* low, *M* medium, *H* high, *NS* nonsailors, *IS* intermediate sailors, *ES* expert sailors

#### Interaction effects

**Fig. 5:**
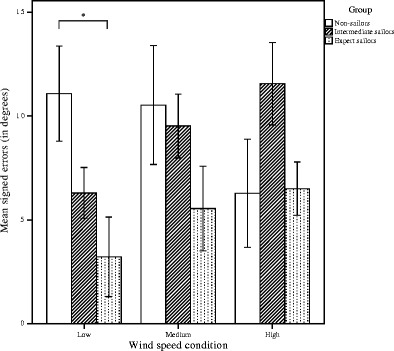
Mean signed errors (MSEs) in degrees (vertical axis) per group, for the three wind conditions (low, medium, high). In the low wind speed condition, expert sailors outperformed nonsailors, *p* = *.*03; vertical bars indicate the standard errors

There was a significant interaction effect for wind direction and wind speed, *F*(30, 450) = 2.4, *p* < .001, *η*_p_^2^ = .14. More-detailed inspection of the data (Table 2) indicated that participants performed with smaller errors for frontal than for rear wind directions primarily when confronted with medium wind speeds—that is, speeds with an average of 3.9 ± 0.9 knots (for all significant post-hoc comparisons, see Table 2). Most importantly, we also found a significant interaction effect between wind speed and group, *F*(4, 30) = 3.6, *p* = *.*017, *η*_p_^2^ = .32. Bonferroni-corrected post-hoc comparisons revealed that for the low wind speed condition, expert sailors (*M* = 3.2°) were significantly more accurate than nonsailors (*M* = 11.1°), *p* = *.*03, 95% CI [0.8°–15.0°] (see Fig. 5). No other interactions were significant.

### Wind speed estimates

**Fig. 6:**
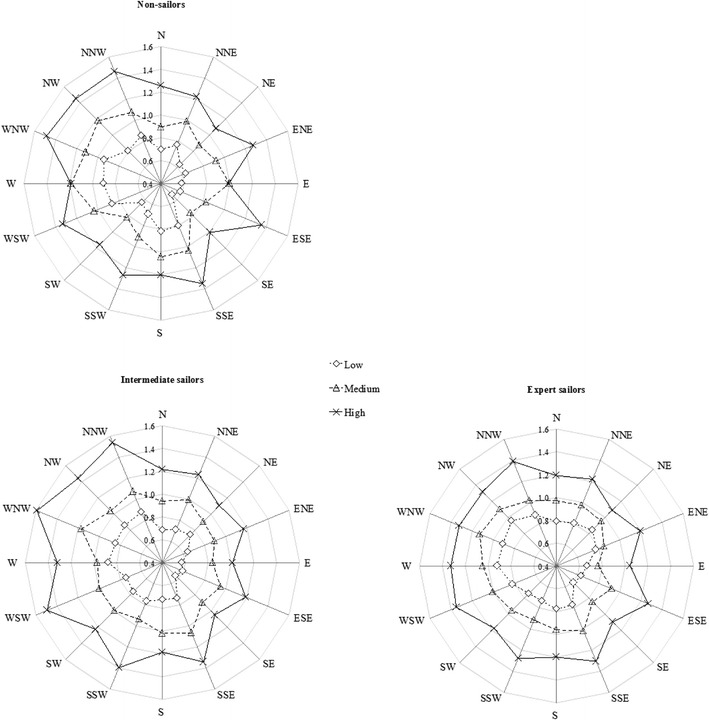
Normalized wind speed estimates per group for the three wind speed conditions (low, medium, high). The estimates are plotted for all 16 compass directions (see Table 1 for the corresponding actual wind directions)

The descriptive data (normalized wind speed estimates) are illustrated in Fig. 6 as polar plots for each group separately.

#### Main effects

The 16 (wind directions) × 3 (wind speed: low, medium, high) × 3 (group: nonsailors, intermediate and expert sailors) mixed-design ANOVA revealed a significant main effect of wind direction on the normalized wind speed estimates, *F*(15, 225) = 27.3, *p* < .001, *η*_p_^2^ = .65. Participants estimated wind stimuli from frontal wind directions to be faster than wind stimuli from the rear (for all significant post-hoc comparisons, see Appendix Table 3). A significant main effect also emerged for wind speed, *F*(2, 30) = 176, *p* < .001, *η*_p_^2^ = .92 (for all significant post-hoc comparisons, see Appendix Table 3). Additionally, we found a significant main effect of group, *F*(2, 15) = 3.8, *p* = *.*048, *η*_p_^2^ = .33 (see Fig. 6). However, Bonferroni-corrected post-hoc comparisons showed no significant interactions.

#### Interaction effects

There was a significant interaction effect between wind direction and wind speed on the normalized wind speed estimates, *F*(30, 450) = 2.7, *p* < .001, *η*_p_^2^ = .16. In general, participants estimated trials from frontal wind directions to be faster than those from rear wind directions for all three wind speed conditions (for all significant post-hoc comparisons, see Appendix Table 3). No other interactions were significant.

#### Dependence on actual wind speed

**Fig. 7:**
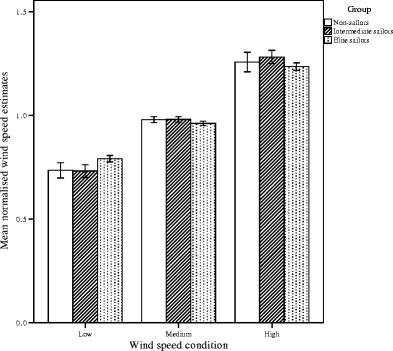
Mean normalized wind speed estimates (vertical axis) per group for the three wind speed conditions (low, medium, high). Vertical bars indicate the standard errors

In Fig. 7, the normalized wind speed estimates are plotted for the three nominal wind speeds, averaged over all wind directions. A power function of the form *f*(*x*) = *c x*^*α*^ was fitted to the normalized wind speed estimates of each group (nonsailors, *α* = 0.65; intermediate sailors, *α* = 0.57; expert sailors, *α* = 0.47) and of each participant separately (range *α* = 0.29–1.02). On average, the *α* of each individual power function (*M* = 0.56, *SE* = 0.05) was significantly smaller than 1 (i.e., a linear relationship), *t*(18) = 8.8, *p* < .001, *r* = .99, indicating a relationship with a decreasing slope. That is, for higher wind speeds, the increase of perceived wind speed as a function of actual wind speed was relatively smaller. We observed no significant difference between groups in the exponents, *F*(2, 15) = 1.0, *p* = *.*39, *η*_p_^2^ = .12.

## Discussion

The aim of this study was to explore expertise effects in cutaneous wind perception. To this end, we presented three groups of participants (i.e., expert sailors, less skilled sailors, and inexperienced controls) with wind stimuli reflecting 16 different wind directions and three different speeds, generated by a wind simulator. Participants rated the wind directions and speeds exclusively on the basis of cutaneous perception. Access to any visual or auditory information was withheld. Most notably, our results showed that the wind direction estimates of expert sailors were significantly more accurate than those of nonsailors when both were presented with low wind speeds.

To specify, whereas in the highest wind speed condition (mean 4.8 knots) no differences were apparent between the three groups with regard to their wind direction estimates, expert sailors were better at discriminating wind directions when higher sensitivity was demanded by the task—that is, when lower wind speeds were presented (mean 3.0 knots). Similar expertise effects have been reported for other haptic tasks. For example, blind people perform better on tactile discrimination tasks than do normally sighted humans (for an overview of expertise in touch perception, see Reuter, Voelcker-Rehage, Vieluf, & Godde, 2012). For blind people, Wong, Gnanakumaran, and Goldreich (2011) suggested that experience-dependent mechanisms cause better perception, and not the loss of sight itself. To the best of our knowledge, ours is the first study to have unraveled expertise effects in the cutaneous perception of wind. It follows that this finding makes a novel contribution to expertise research in general, and to our understanding of perceptual expertise in particular; at the same time, and given the exploratory nature of this study, the results raise an interesting question for the future.

This question is, Why did experts only outperform novices in rating wind direction with wind speeds of about 3 knots, but not with higher wind intensities? Though more research will be necessary to underpin our speculations, we argue that a possible explanation may be that with developing expertise (and the accumulation of experience), the accuracy of wind perception may be modulated—that is, may be enhanced. In fact, it seems that with wind speeds close to 3 knots, experts sailors who are trained and have accumulated significant experience at perceiving wind directions at various wind intensities benefit from a higher sensitivity to cutaneously perceived stimuli, whereas control participants are not sensitive enough to correctly perceive wind directions with such low wind speeds. Admittedly, the wind intensities we were able to reliably produce using the wind simulator were rather low, and the range of simulated wind speeds was rather small (i.e., between 3 and 4.8 knots, as compared to the winds of typically 5–25 knots experienced during sailing regattas). It could be that by chance we detected the wind speed threshold that allowed us to differentiate between experts (i.e., sailors) and laymen with respect to their ability to accurately perceive wind directions.

If our reasoning is sound, one might argue that we should have also found group differences for the normalized wind speed estimates in the three simulated wind speed conditions, or at least in the lowest wind speed condition. Our results indeed showed this main effect of group, yet post-hoc pairwise comparisons yielded no differences between the individual groups. Though this may seem to be a contradiction at first sight, we argue that it is not: Expertise in sailing, particularly in relation to wind perception, first and foremost is characterized by detecting the right wind direction in order to steer the boat optimally (for more information on sailing theory, see Davidson, 2009). This is true regardless of the intensity of the wind. It follows that the ability to accurately perceive wind speeds (in itself), even though it may co-develop as a subserving capability, does not have the same impact on sailors’ performance, and hence may be of lower priority. Clearly, this theory is somewhat speculative. We do not know what may or may not happen at either lower or significantly higher wind speeds, and we prefer not to overinterpret our findings, but rather wish to call for more research examining a broader range of wind intensities and larger sample sizes in order to test our ideas.

As regards the relationship between the normalized estimates and actual wind speed, we found a good fit using a power function. In other studies, power function exponents have been found to differ for various perceptual continua. For example, concerning other aspects of tactual perception, the exponent for tactual hardness perception was smaller than 1, namely 0.8 (Harper & Stevens, 1964), whereas that for tactual roughness perception was larger than 1, being 1.5 (Stevens & Harris, 1962). In the present study, an exponent smaller than 1 was found, implying that the slope of the relationship—between perceived and physical intensity—decreases with increasing wind speed. This indicates that wind speed differences in the low range are perceptually magnified, relative to differences in the higher ranges. This might be advantageous for sailors because, especially in regattas sailed with low wind speeds, a correct estimate of the wind speed is essential for the accompanying trim of the sails.

In addition to the finding that expert sailors outperformed nonsailors in wind perception with low wind speeds, our results revealed that wind direction estimates also differed depending on wind speeds, and vice versa—that is, wind speed estimates differed depending on wind directions (see Table 2 and Appendix Table 3). Post-hoc comparisons seem to indicate that participants rated wind directions presented from the front more accurately than those presented from the back. This was particularly the case with medium wind speeds (mean 3.9 knots). Participants were instructed to face the video screen in front of them. However, we did not systematically measure or record head movements in our experimental setup. Therefore, one might argue that head movements (i.e., kinesthetic information) may have facilitated more accurate cutaneous wind perception, especially for the frontal wind directions. If this were true, then we would expect to find an interaction effect between group and wind direction (divided into frontal and rear wind directions). However, additional analyses^1^ did not reveal such an interaction, and hence we deem it unlikely that head movements can serve as an explanation for the expertise effect reported. As a suggestion, future research might explore alternative response measures, such as participants pointing their arm in the perceived wind direction while sitting in a rotatable chair. Likewise, participants perceived wind stimuli from frontal directions as being more intense than those from rear directions. These findings certainly do not come as a surprise, but rather show that the wind simulator provides a valid and reliable setup for gathering wind direction and speed estimates.

Finally, this has been the first study to show expertise effects in cutaneous wind perception. We further showed that experts in sailing are more accurate in estimating wind directions with low wind speeds. It is important to note, however, that expertise effects in cutaneous wind perception are not the only distinctions between expert sailors and novices that may account for experts’ superior performance in the field. Sailing expertise also relates to better use of visual information and efficient motor behavior. For example, in an in-situ experiment, Pluijms, Cañal-Bruland, Hoozemans, and Savelsbergh (2015) recently showed that better performance during the windward mark rounding in sailing was related to gazing more to the tangent point during the actual rounding. With respect to movement behavior and boat control, superior performance was associated with release of the trimming lines close to rounding the mark and approaching the mark with little heel. In other words, in sailing—but also in many other domains, such as car driving or aviation—the integration of multiple sources of sensory information is crucial to performance. In this regard, the present study provides initial evidence that cutaneous perception contributes to expert performance in sailing. Accordingly, future research will need to further explore the degree to which the pickup of information via the individual senses adds to performance, and whether experts may differ from their less-experienced counterparts in integrating multisensory information to guide their actions (Gray, 2008). Capitalizing on the results of the present study, a training study would amend our insights into these processes.

## Figures and Tables

**Fig. 8:**
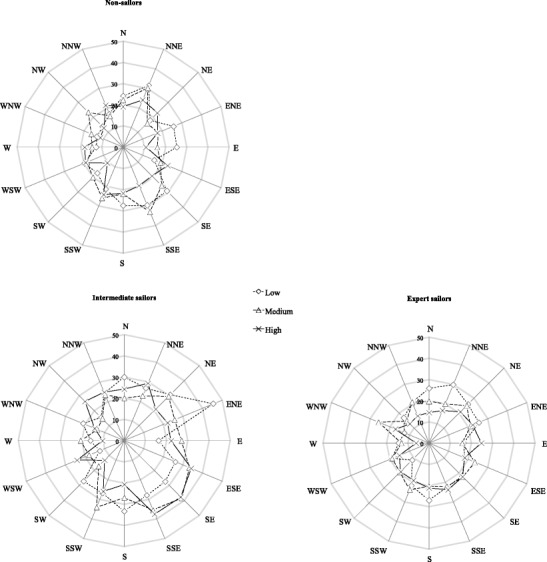
Mean absolute errors (MAEs) in degrees for the three wind speed conditions (low, medium, high) and 16 compass directions (see Table 1 for the corresponding actual wind directions). Upper left: Nonsailors. Bottom left: Intermediate sailors. Bottom right: Expert sailors

## Appendix A

Note that since the experiment was designed to measure accuracy, our main analysis was based on the MSEs. However, for the sake of completeness, we also examined potential group differences in mean absolute errors (MAEs). A 16 (wind directions) × 3 (wind speed: low, medium, high) × 3 (group: nonsailors, intermediate and expert sailors) mixed-design ANOVA on the MAEs revealed a significant main effect of group, *F*(2, 15) = 4.1, *p* = *.*038, *η*_p_^2^ = .35 (see Fig. 8 for the polar plots). Bonferroni-corrected pairwise comparisons showed no significant differences between groups. No interactions between the factor Group and the wind directions or wind speeds were significant.

## Appendix B

**Table 3:** Significant post-hoc pairwise comparisons, Bonferroni corrected, of normalized wind speed estimates (NWSE) for the wind direction estimates, with the 16 Compass Directions (CD) and three Wind Speed Conditions (WSC) as within-subjects factors

Wind Direction						
	CD	NSWE	CD	NWSE	*p*	95% CI for difference
	N	0.96	SE	0.82	<.01	0.03–0.24
	N	0.96	WSW	1.07	.01	0.02–0.21
	N	0.96	W	1.09	.03	0.00–0.24
	N	0.96	WNW	1.17	<.01	0.06–0.36
	N	0.96	NW	1.13	<.001	0.10–0.24
	N	0.96	NNW	1.13	<.001	0.10–0.24
	NNE	0.98	E	0.84	<.01	0.03–0.26
	NNE	0.98	SE	0.82	<.001	0.07–0.25
	NNE	0.98	WNW	1.17	<.01	0.04–0.33
	NNE	0.98	NW	1.13	<.01	0.04–0.25
	NNE	0.98	NNW	1.13	<.001	0.07–0.23
	NE	0.91	WSW	1.07	.03	0.01–0.31
	NE	0.91	WNW	1.17	<.001	0.09–0.42
	NE	0.91	NW	1.13	<.01	0.06–0.37
	NE	0.91	NNW	1.13	<.001	0.10–0.33
	ENE	0.93	SE	0.82	<.01	0.02–0.18
	ENE	0.93	SSE	1.05	.01	0.02–0.23
	ENE	0.93	WSW	1.07	.02	0.02–0.27
	ENE	0.93	WNW	1.17	<.01	0.08–0.40
	ENE	0.93	NW	1.13	<.001	0.12–0.29
	ENE	0.93	NNW	1.13	<.001	0.11–0.30
	E	0.84	SSE	1.05	<.001	0.09–0.32
	E	0.84	S	0.99	<.01	0.05–0.26
	E	0.84	WSW	1.07	<.001	0.12–0.34
	E	0.84	W	1.09	<.01	0.06–0.43
	E	0.84	WNW	1.17	<.001	0.25–0.41
	E	0.84	NW	1.13	<.001	0.15–0.43
	E	0.84	NNW	1.13	<.001	0.20–0.38
	ESE	0.92	SE	0.82	.01	0.02–0.18
	ESE	0.92	SSE	1.05	.04	0.00–0.25
	ESE	0.92	WSW	1.07	.02	0.01–0.29
	ESE	0.92	WNW	1.17	<.001	0.13–0.36
	ESE	0.92	NW	1.13	<.001	0.08–0.33
	ESE	0.92	NNW	1.13	<.001	0.12–0.29
	SE	0.82	SSE	1.05	<.001	0.10–0.35
	SE	0.82	S	0.99	<.01	0.04–0.30
	SE	0.82	SSW	0.99	<.01	0.04–0.28
	SE	0.82	SW	0.94	<.001	0.05–0.18
	SE	0.82	WSW	1.07	<.001	0.12–0.37
	SE	0.82	W	1.09	<.01	0.08–0.44
	SE	0.82	WNW	1.17	<.001	0.20–0.49
	SE	0.82	NW	1.13	<.001	0.19–0.42
	SE	0.82	NNW	1.13	<.001	0.20–0.42
	S	0.99	WNW	1.17	<.01	0.05–0.30
	S	0.99	NW	1.13	.03	0.01–0.27
	S	0.99	NNW	1.13	<.01	0.04–0.24
	SSW	0.99	WNW	1.17	<.05	0.00–0.36
	SSW	0.99	NW	1.13	<.001	0.06–0.23
	SSW	0.99	NNW	1.13	<.01	0.04–0.24
	SW	0.99	WNW	1.17	<.01	0.06–0.39
	SW	0.99	NW	1.13	<.01	0.07–0.31
	SW	0.99	NNW	1.13	<.001	0.07–0.31
Wind Speed						
	WSC	NSWE	WSC	NWSE	*p*	95% CI for difference
	L	0.75	M	0.97	<.001	0.18–0.27
	L	0.75	H	1.26	<.001	0.41–0.60
	M	0.97	H	1.26	<.001	0.22–0.35
Wind Direction × Wind Speed						
WSC	CD	NSWE	CD	NWSE	*p*	95% CI for difference
L	N	0.73	W	0.90	<.01	0.03–0.31
L	N	0.73	NW	0.88	.02	0.01–0.29
L	NNE	0.75	ESE	0.60	.01	0.21–0.28
L	NNE	0.75	SE	0.57	<.01	0.05–0.31
L	NE	0.73	SE	0.57	.01	0.02–0.30
L	ENE	0.67	W	0.90	.03	0.01–0.44
L	ENE	0.67	NW	0.88	<.01	0.04–0.37
L	E	0.63	W	0.90	<.01	0.07–0.46
L	E	0.63	WNW	0.90	<.01	0.08–0.45
L	E	0.63	NW	0.88	.02	0.02–0.46
L	E	0.63	NNW	0.87	<.001	0.10–0.37
L	ESE	0.60	WSW	0.81	<.01	0.05–0.36
L	ESE	0.60	W	0.90	<.001	0.11–0.48
L	ESE	0.60	WNW	0.90	<.001	0.12–0.47
L	ESE	0.60	NW	0.88	<.01	0.09–0.46
L	ESE	0.60	NNW	0.87	<.01	0.11–0.42
L	SE	0.57	SW	0.71	.03	0.01–0.27
L	SE	0.57	W	0.90	<.01	0.13–0.53
L	SE	0.57	WNW	0.90	<.01	0.11–0.54
L	SE	0.57	NW	0.88	<.001	0.12–0.48
L	SE	0.57	NNW	0.87	<.001	0.11–0.49
L	S	0.77	WNW	0.90	.03	0.01–0.24
L	SW	0.71	W	0.90	.02	0.02–0.35
L	SW	0.71	NW	0.88	.03	0.01–0.32
M	N	0.94	WNW	1.14	.02	0.02–0.38
M	NE	0.98	WNW	1.14	<.01	0.06–0.39
M	NE	0.98	NW	1.11	.04	0.11–0.38
M	NE	0.98	NNW	1.06	.02	0.01–0.27
M	ENE	0.89	SSE	1.04	.03	0.01–0.29
M	ENE	0.89	WNW	1.14	<.01	0.05–0.45
M	E	0.87	WNW	1.14	<.01	0.06–0.48
M	ESE	0.89	WNW	1.14	<.001	0.10–0.40
M	SE	0.85	S	1.01	<.01	0.03–0.29
M	SE	0.85	WSW	1.01	<.01	0.04–0.29
M	SE	0.85	WNW	1.14	<.001	0.11–0.47
M	SE	0.85	NW	1.11	.01	0.03–0.49
M	SE	0.85	NNW	1.06	.03	0.01–0.40
M	SSW	0.92	NW	1.11	<.001	0.08–0.30
M	SW	0.92	WNW	1.14	<.01	0.06–0.37
M	WSW	1.01	WNW	1.14	.02	0.01–0.24
H	N	1.22	E	1.02	<.001	0.07–0.34
H	N	1.22	SE	1.05	<.001	0.07–0.27
H	N	1.22	WNW	1.47	.01	0.04–0.46
H	N	1.22	NW	1.40	<.001	0.09–0.28
H	N	1.22	NNW	1.46	<.01	0.06–0.42
H	NNE	1.23	E	1.02	.03	0.01–0.35
H	NNE	1.23	SE	1.05	<.05	0.00–0.35
H	NNE	1.23	NW	1.40	.02	0.02–0.34
H	NNE	1.23	NNW	1.46	<.01	0.04–0.44
H	NE	1.09	SSE	1.33	.02	0.02–0.46
H	NE	1.09	WSW	1.39	<.01	0.07–0.53
H	NE	1.09	WNW	1.47	<.001	0.19–0.57
H	NE	1.09	NW	1.40	<.001	0.15–0.47
H	NE	1.09	NNW	1.46	<.001	0.15–0.60
H	ENE	1.21	E	1.02	.04	0.01–0.39
H	ENE	1.21	SE	1.05	.04	0.00–0.32
H	ENE	1.21	WNW	1.47	.03	0.01–0.50
H	ENE	1.21	NW	1.40	<.01	0.05–0.33
H	ENE	1.21	NNW	1.46	.01	0.03–0.47
H	E	1.02	SSE	1.33	<.01	0.06–0.57
H	E	1.02	SSW	1.31	<.01	0.06–0.52
H	E	1.02	WSW	1.39	<.001	0.16–0.59
H	E	1.02	W	1.29	.04	0.01–0.55
H	E	1.02	WNW	1.47	<.001	0.25–0.66
H	E	1.02	NW	1.40	<.001	0.21–0.56
H	E	1.02	NNW	1.46	<.001	0.24–0.66
H	ESE	1.28	SE	1.05	.02	0.19–0.43
H	ESE	1.28	NNW	1.46	.03	0.01–0.37
H	SE	1.28	SSE	1.33	<.01	0.07–0.50
H	SE	1.28	SSW	1.31	<.01	0.06–0.46
H	SE	1.28	SW	1.19	.02	0.01–0.26
H	SE	1.28	WSW	1.39	<.001	0.13–0.56
H	SE	1.28	W	1.29	.04	0.01–0.48
H	SE	1.28	WNW	1.47	<.001	0.22–0.62
H	SE	1.28	NW	1.40	<.001	0.23–0.48
H	SE	1.28	NNW	1.46	<.001	0.20–0.62
H	S	1.20	NW	1.40	.01	0.03–0.39
H	S	1.20	NNW	1.46	.03	0.01–0.52
H	SW	1.19	WNW	1.47	<.01	0.06–0.51
H	SW	1.19	NW	1.40	<.001	0.09–0.34
H	SW	1.19	NNW	1.46	<.01	0.09–0.47

*L* low, *M* medium, *H* high, *NS* nonsailors, *IS* intermediate sailors, *ES* expert sailors

## Footnotes

1 We divided the 16 wind directions into two wind direction clusters based on the actual wind directions (i.e., frontal and rear wind directions; see Table 1). A 2 (wind direction cluster) × 3 (wind speed: low, medium, high) × 3 (group: nonsailors, intermediate and expert sailors) mixed-design ANOVA on the MSEs revealed no significant interaction effect between wind direction cluster and group, *F*(2, 15) = 0.9, *p* = .45. We found a significant three-way interaction between wind direction cluster, wind speed condition, and group, *F*(4, 30) = 3.2, *p* = .028. However, Bonferroni-corrected post-hoc comparisons showed no significant interactions between group and wind speed condition for either the frontal or the rear wind direction cluster.
